# Supplementary material for: Temporal Gene Expression in Apical Culms Shows Early Changes in Cell Wall Biosynthesis Genes in Sugarcane
Source: Front Plant Sci. 2021 Dec 13;12:736797. doi: 10.3389/fpls.2021.736797 (PMC8710541; doi:10.3389/fpls.2021.736797)
Supplement: Supplementary file 5 [file Image_1.PDF]

frontiers

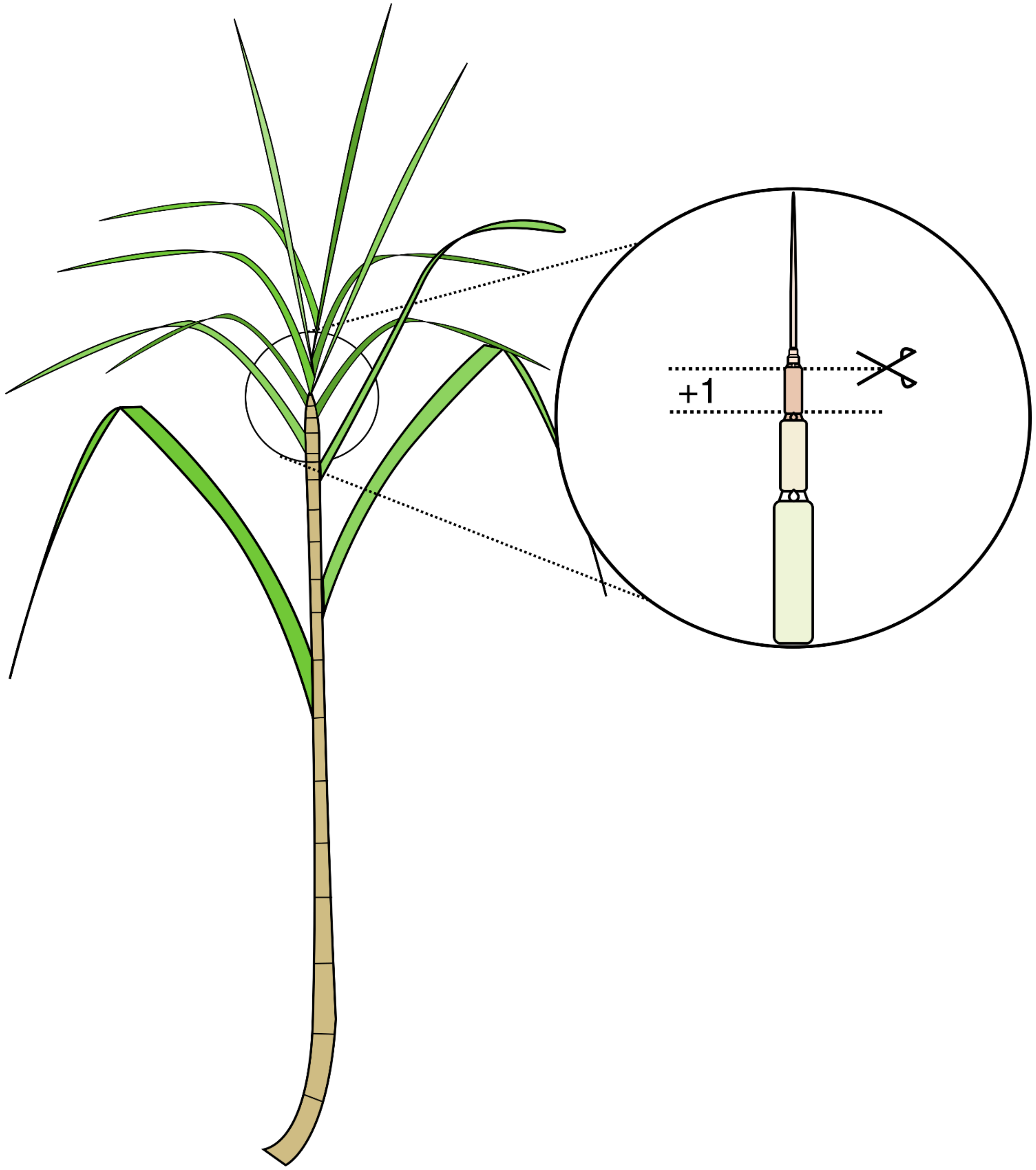

Supplementary Figure 1. Schematic representation of the sampled sugarcane internode +1 harvested for RNA-seq.
