# Supplementary material for: Temporal Gene Expression in Apical Culms Shows Early Changes in Cell Wall Biosynthesis Genes in Sugarcane
Source: Front Plant Sci. 2021 Dec 13;12:736797. doi: 10.3389/fpls.2021.736797 (PMC8710541; doi:10.3389/fpls.2021.736797)
Supplement: Supplementary file 6 [file Image_2.PDF]

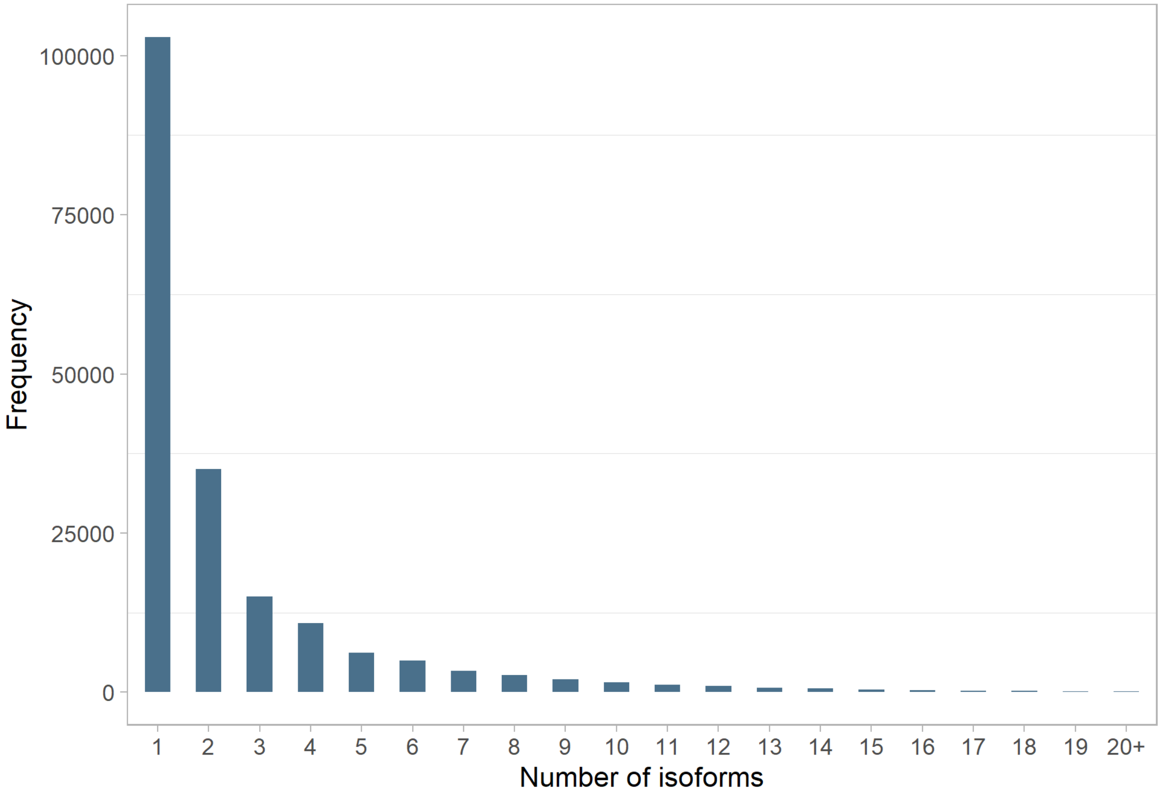

Supplementary Figure 2. Distribution of the number of isoforms per unigene in the de novo assembly of the sugarcane transcriptome.
