# Supplementary material for: Temporal Gene Expression in Apical Culms Shows Early Changes in Cell Wall Biosynthesis Genes in Sugarcane
Source: Front Plant Sci. 2021 Dec 13;12:736797. doi: 10.3389/fpls.2021.736797 (PMC8710541; doi:10.3389/fpls.2021.736797)
Supplement: Supplementary file 7 [file Image_3.PDF]

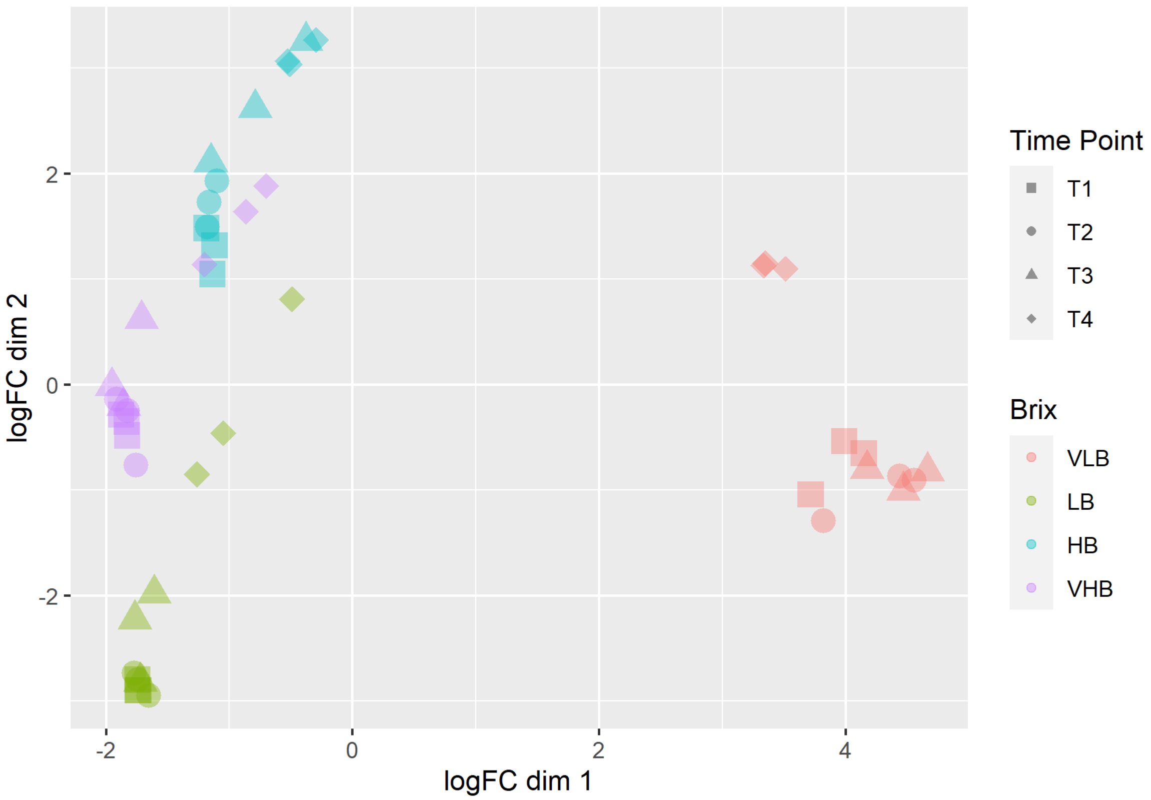

Supplementary Figure 3. Multidimensional scaling (MDS) plot based on gene expression profiles of immature sugarcane culms. The figure shows grouping of samples from the same genotype, with prominent separation of IN84-58 samples (very low ºBrix) from the rest. Samples from 12-month-old (T4) are more clearly separated from the other time points. VLB: very low ºBrix, LB: low ºBrix, HB: high ºBrix and VHB: very high ºBrix. T1: 6-month-old, T2: 8-month-old, T3: 10-month-old and T4: 12-month-old.
