# Supplementary material for: Temporal Gene Expression in Apical Culms Shows Early Changes in Cell Wall Biosynthesis Genes in Sugarcane
Source: Front Plant Sci. 2021 Dec 13;12:736797. doi: 10.3389/fpls.2021.736797 (PMC8710541; doi:10.3389/fpls.2021.736797)
Supplement: Supplementary file 8 [file Image_4.PDF]

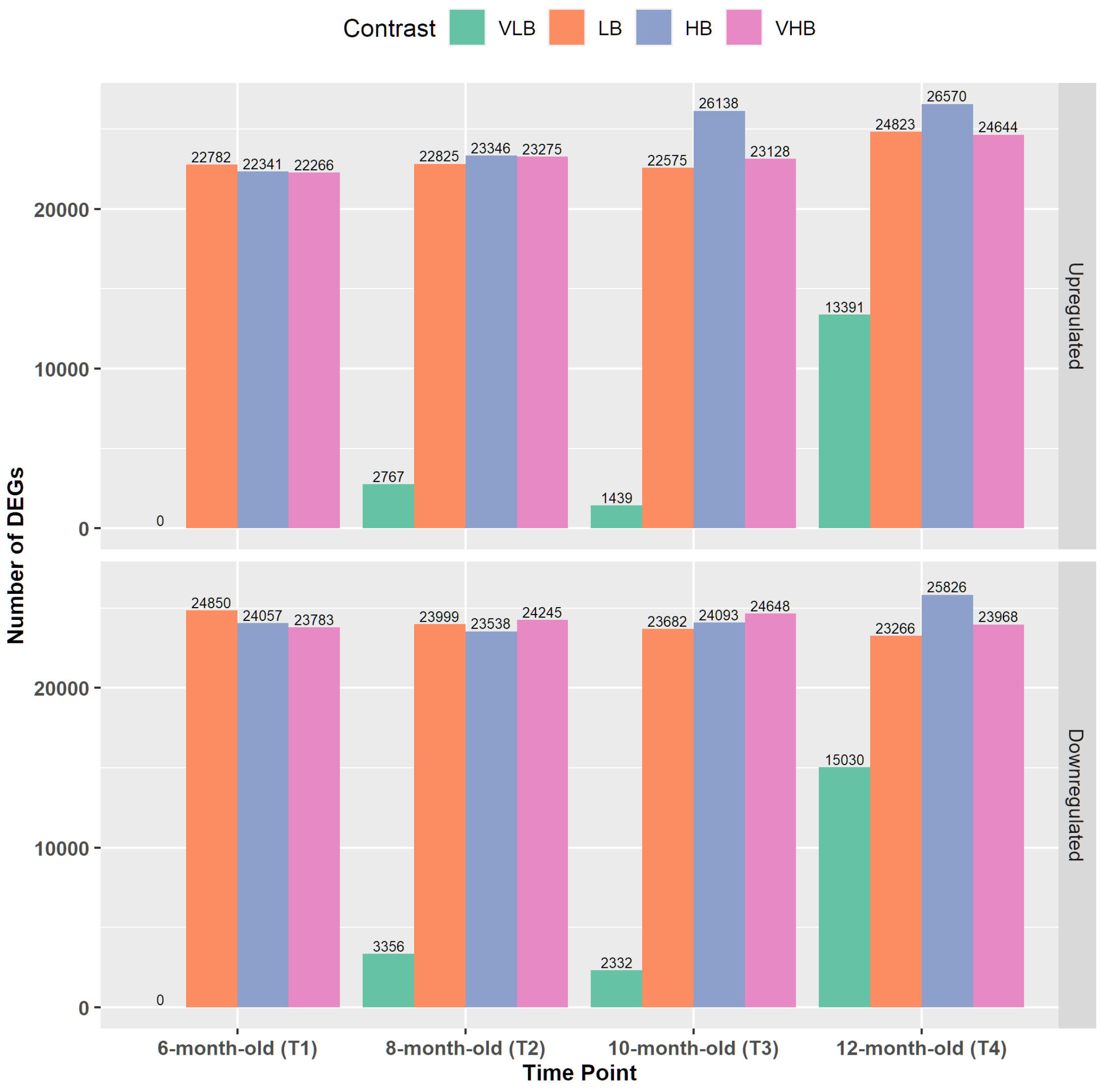

Supplementary Figure 4. Numbers of differentially expressed genes (DEGs) per genotype. The x axis shows the age of plants (in months). Upregulated or downregulated genes are presented separately. VLB: very low ºBrix, LB: low ºBrix, HB: high ºBrix, VHB: very high ºBrix. Treatment group VLB at T1 was used as a reference for pairwise tests involving combinations of genotype and time points.
