# Supplementary material for: Temporal Gene Expression in Apical Culms Shows Early Changes in Cell Wall Biosynthesis Genes in Sugarcane
Source: Front Plant Sci. 2021 Dec 13;12:736797. doi: 10.3389/fpls.2021.736797 (PMC8710541; doi:10.3389/fpls.2021.736797)
Supplement: Supplementary file 10 [file Image_6.PDF]

A)

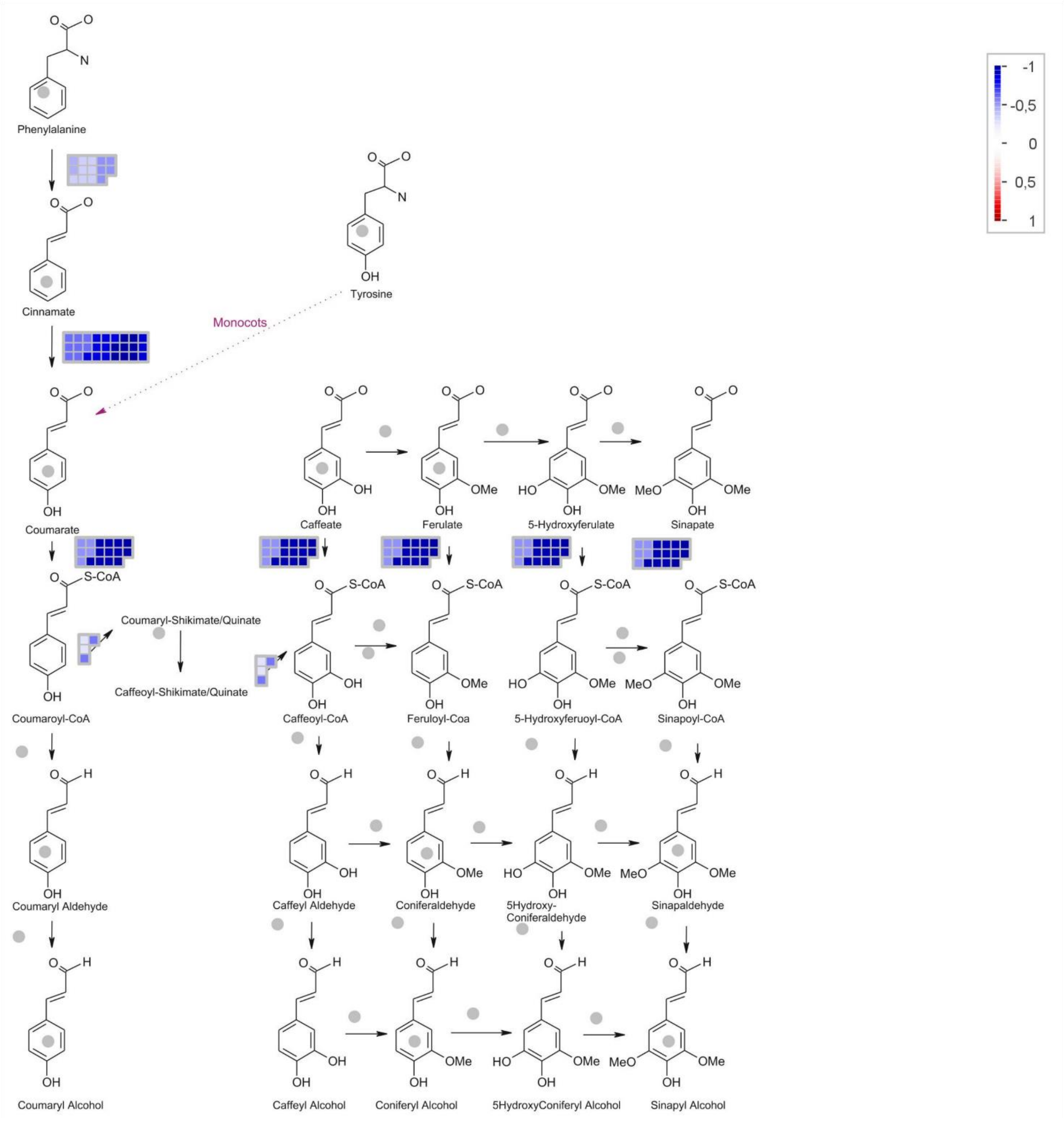

Phenylalanine
Cinnamate
Tyrosine
Monocots
Coumarate
Caffeate
Ferulate
5-Hydroxyferulate
Sinapate
Coumaroyl-CoA
Coumaryl-Shikimate/Quinate
Caffeoyl-Shikimate/Quinate
Caffeoyl-CoA
Feruloyl-Coa
5-Hydroxyferuoyl-CoA
Sinapoyl-CoA
Coumaryl Aldehyde
Caffeyl Aldehyde
Coniferaldehyde
5Hydroxy-Coniferaldehyde
Sinapaldehyde
Coumaryl Alcohol
Caffeyl Alcohol
Coniferyl Alcohol
5HydroxyConiferyl Alcohol
Sinapyl Alcohol
-1
-0,5
0
0,5
1

B)

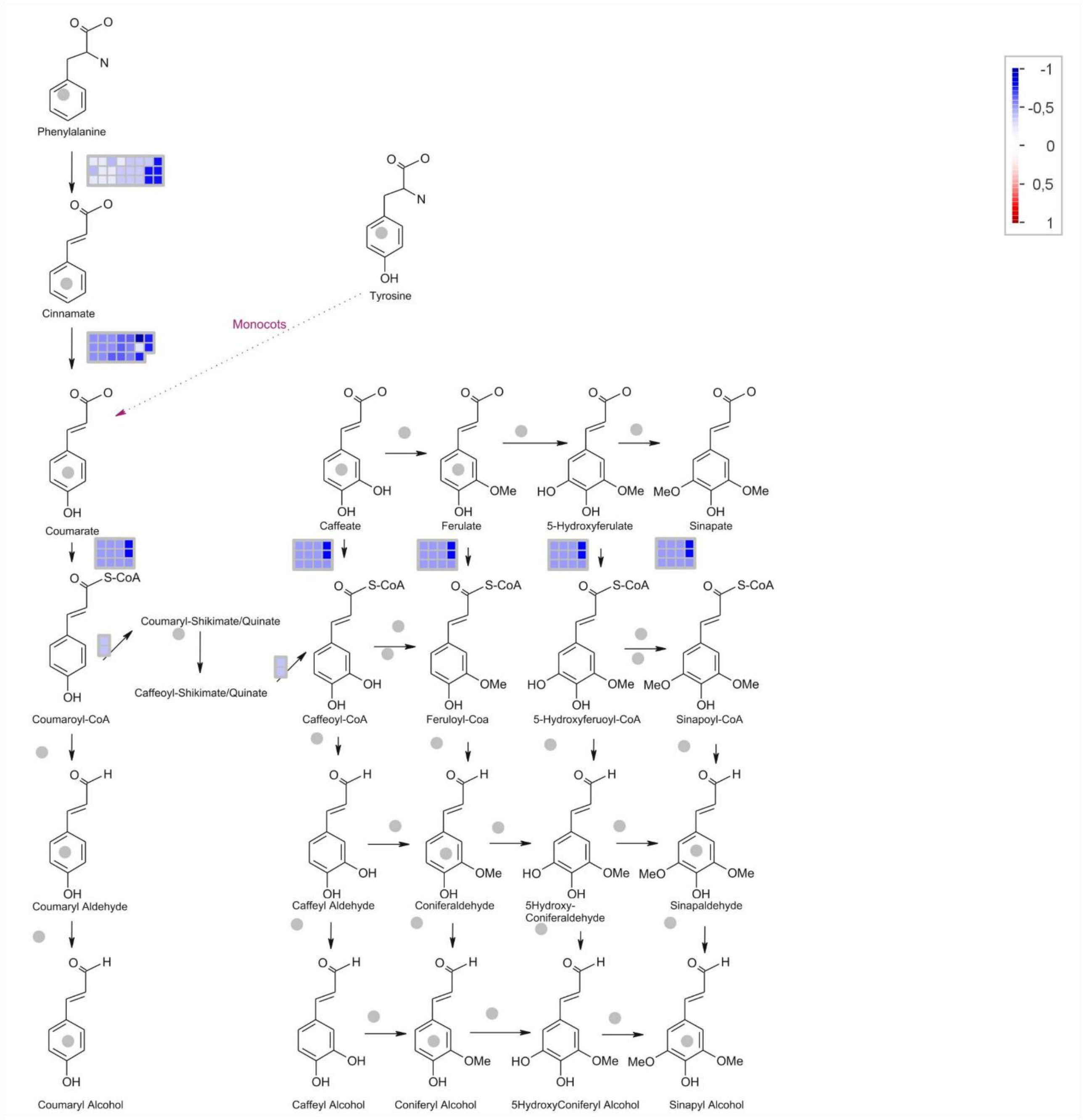
Phenylalanine
Cinnamate
Tyrosine
Monocots
Coumarate
Caffeate
Ferulate
5-Hydroxyferulate
Sinapate
Coumaroyl-CoA
Coumaryl-Shikimate/Quinate
Caffeoyl-Shikimate/Quinate
Caffeoyl-CoA
Feruloyl-Coa
5-Hydroxyferuoyl-CoA
Sinapoyl-CoA
Coumaryl Aldehyde
Caffeyl Aldehyde
Coniferaldehyde
5Hydroxy-
Coniferaldehyde
Sinapaldehyde
Coumaryl Alcohol
Caffeyl Alcohol
Coniferyl Alcohol
5HydroxyConiferyl Alcohol
Sinapyl Alcohol
-1
-0,5
0
0,5
1

C)

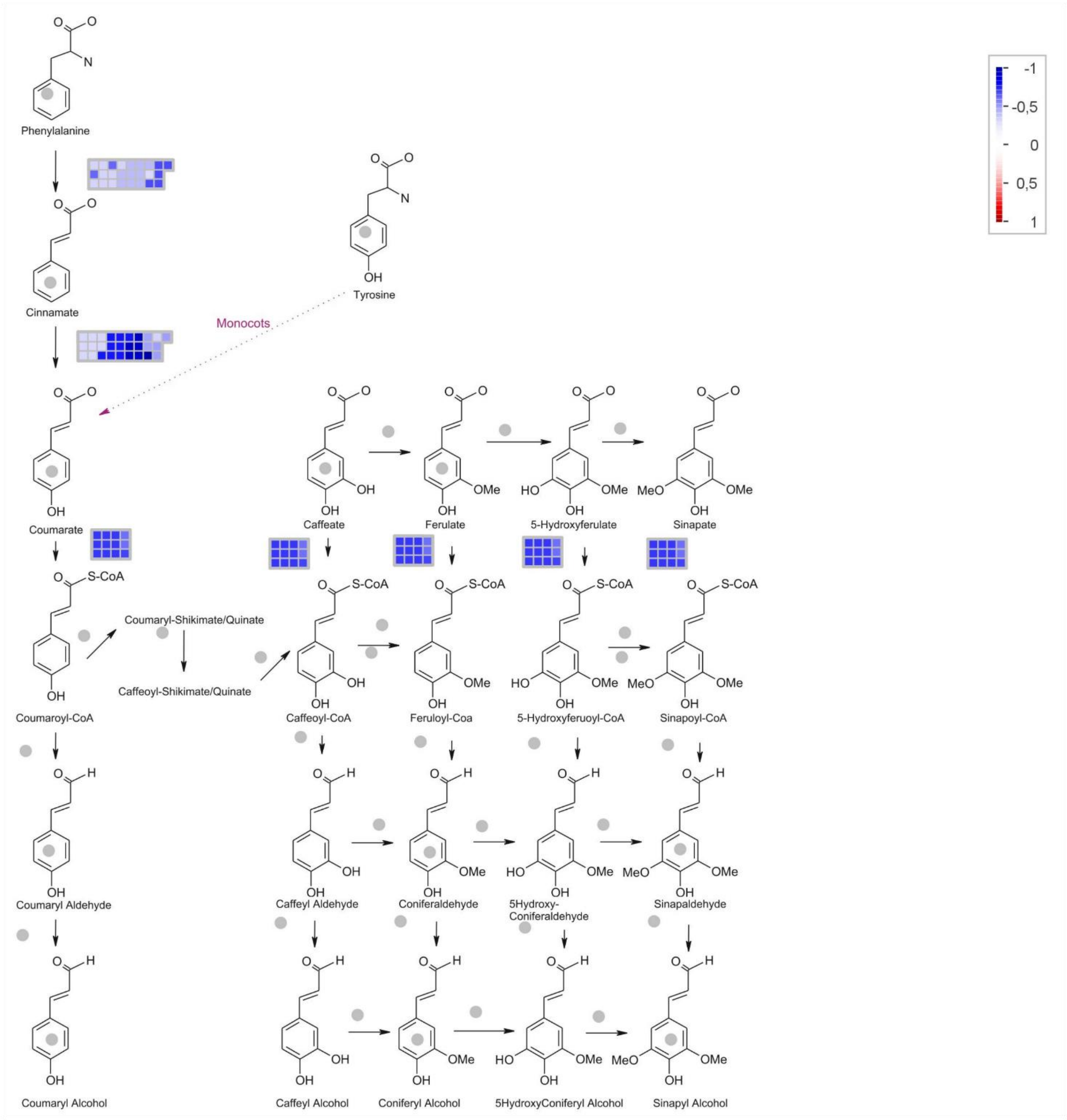

Supplementary Figure 6. MapMan expression profiles in the phenylpropanoid pathway considering main effects between genotypes. Each square represents a gene involved in the pathway and the colors denote the corresponding log fold change in comparison to the reference (VLB). The blue, white and red colors characterize downregulated, non-differentially expressed and upregulated genes, respectively. (A) VHB vs VLB contrast; (B) HB vs VLB contrast; (C) LB vs VLB contrast. VHB: very high ºBrix, HB: high ºBrix; LB: low ºBrix; and VLB: very low ºBrix.
