# Supplementary material for: Temporal Gene Expression in Apical Culms Shows Early Changes in Cell Wall Biosynthesis Genes in Sugarcane
Source: Front Plant Sci. 2021 Dec 13;12:736797. doi: 10.3389/fpls.2021.736797 (PMC8710541; doi:10.3389/fpls.2021.736797)
Supplement: Supplementary file 11 [file Image_7.PDF]

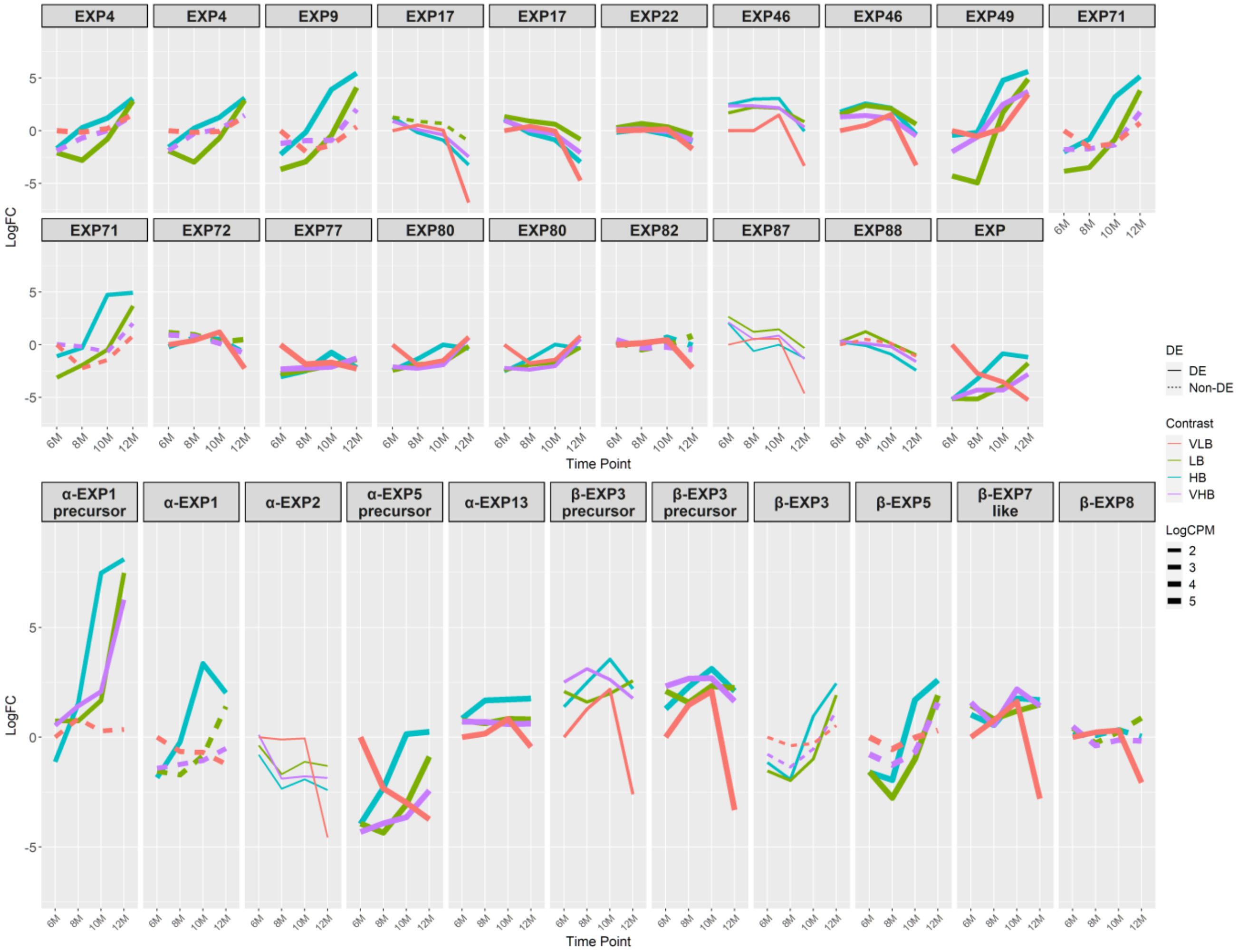

Supplementary Figure 7. Expression profiles of genes of the expansin family. The x axis shows the age of plants (in months). The y axis corresponds to the log fold change in comparison to the very low ºBrix (VLB) genotype in T1 (6-month-old plants). Each line represents a gene and the solid lines indicate genes with significant differential expression in at least at one time point, while dashed lines represent genes with no differential expression for a given genotype. The line width indicates the average expression level of each gene, with more highly expressed genes thicker. EXP: expansin. VHB: very high ºBrix, HB: high ºBrix; LB: low ºBrix; and VLB: very low ºBrix.
