# Supplementary material for: Temporal Gene Expression in Apical Culms Shows Early Changes in Cell Wall Biosynthesis Genes in Sugarcane
Source: Front Plant Sci. 2021 Dec 13;12:736797. doi: 10.3389/fpls.2021.736797 (PMC8710541; doi:10.3389/fpls.2021.736797)
Supplement: Supplementary file 12 [file Image_8.PDF]

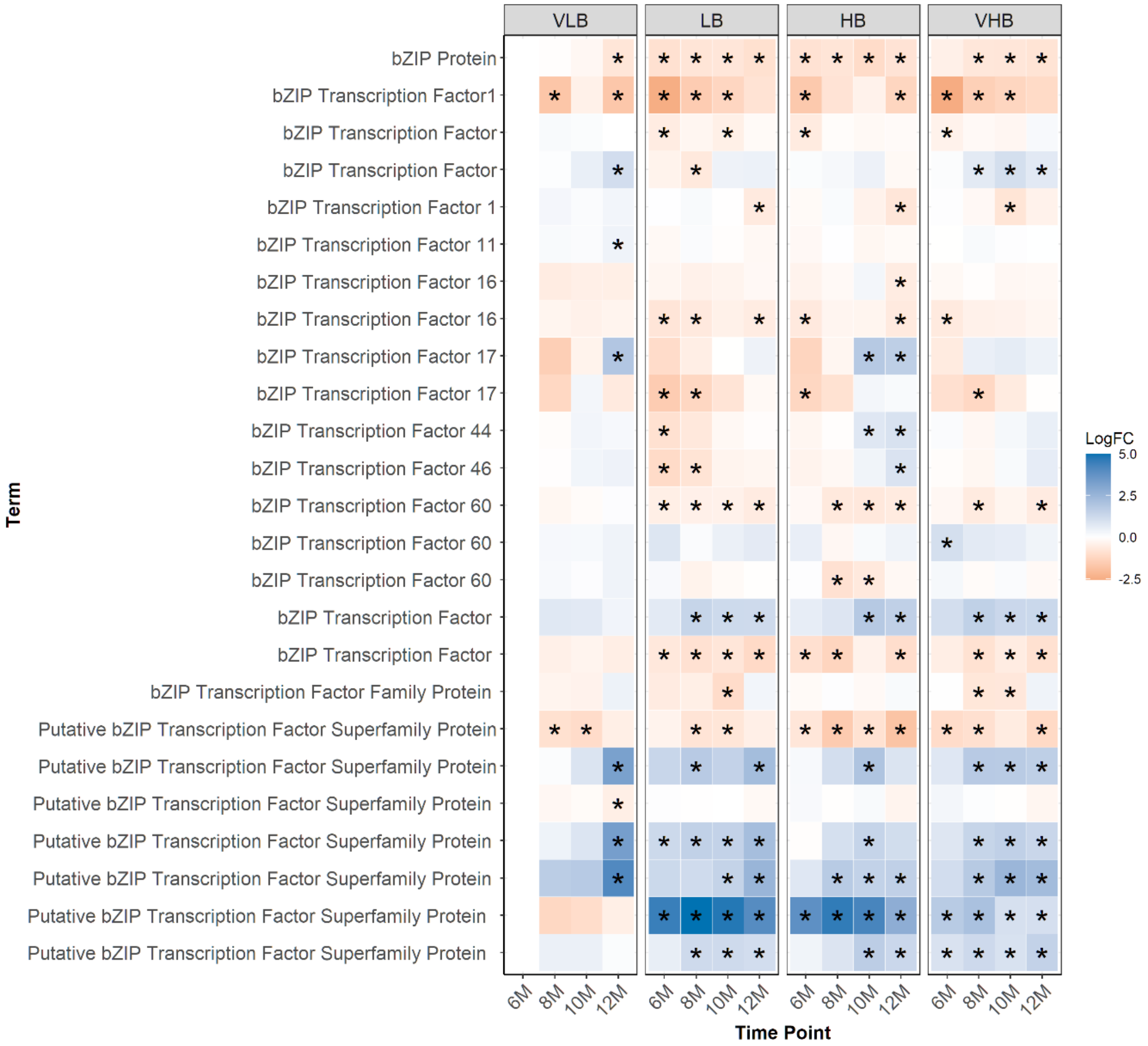

Supplementary Figure 8. Expression profiles of transcription factors of the bZIP family. Asterisks indicate genes with differential expression for a particular combination of genotype and time point. The age of plants in the x axis is shown in months. VLB: very low ºBrix, LB: low ºBrix, HB: high ºBrix and VHB: very high ºBrix. All tests considered 6-month-old VLB as a reference group.
