# Supplementary material for: Temporal Gene Expression in Apical Culms Shows Early Changes in Cell Wall Biosynthesis Genes in Sugarcane
Source: Front Plant Sci. 2021 Dec 13;12:736797. doi: 10.3389/fpls.2021.736797 (PMC8710541; doi:10.3389/fpls.2021.736797)
Supplement: Supplementary file 13 [file Image_9.PDF]

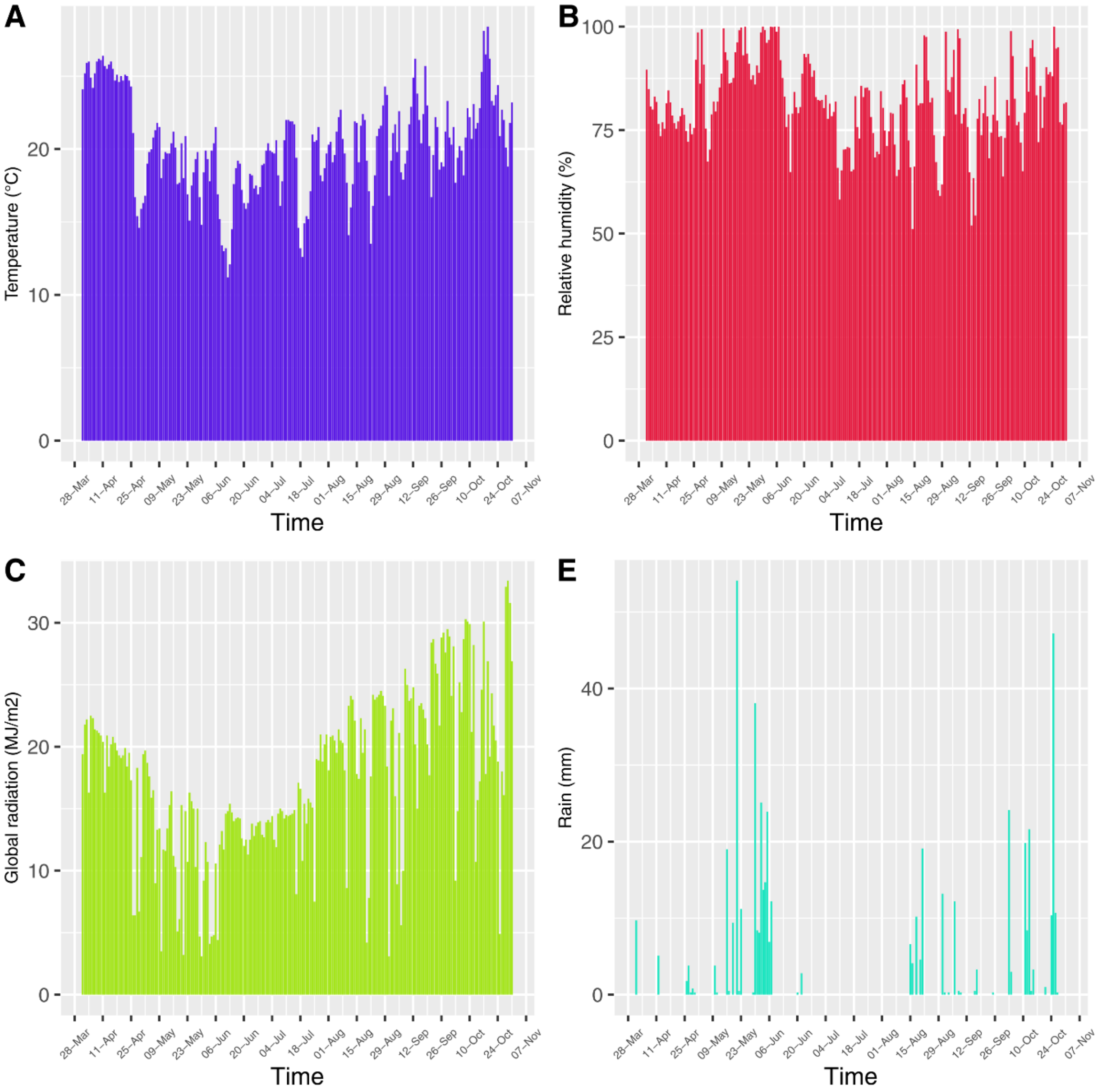

Supplementary Figure 9. Seasonal variation in the field in the period from Abril to October of 2016 obtained from the Automatic Weather Station (AWS) at UFSCar, Araras, SP, Brazil. A) Average temperature (°C). B) Average relative air humidity (%). C) Global solar radiation (MJ/m2). D) Total rain (mm).
